# Outcomes of resection for rectal cancer in India: The impact of the double stapling technique

**DOI:** 10.1186/1477-7819-5-35

**Published:** 2007-03-21

**Authors:** Shailesh V Shrikhande, Rajesh R Saoji, Savio G Barreto, Anagha C Kakade, Stephen D Waterford, Sanjay B Ahire, Fahim M Goliwale, Parul J Shukla

**Affiliations:** 1 Gastrointestinal Surgical Oncology, Tata Memorial Hospital, Mumbai, India; 2 Clinical Research Secretariat, Tata Memorial Hospital, Mumbai, India

## Abstract

**Background:**

The introduction of circular staplers into colorectal surgery has revolutionized anastomotic techniques stretching the limits of sphincter preservation. Data on the double-stapling technique (DST) has been widely published in the West where the incidence of colorectal cancer is high. However studies using this technique and their results, in the Indian scenario, as well as the rest of Asia, have been few and far between.

**Aim:**

To evaluate the feasibility of the DST in Indian patients with low rectal cancers and assess its impact on anastomotic leak rates, covering colostomy rates, level of resection and morbidity in patients undergoing low anterior resection (LAR).

**Methods:**

A comparative analysis was performed between retrospectively acquired data on 78 patients (mean age 53.2 ± 13.5 years) undergoing LAR with the single-stapling technique (SST) (between January 1999 and December 2001) and prospective data acquired on 138 LARs (mean age 50.3 ± 13.9 years) performed using the DST (between January 2003 – December 2005).

**Results:**

A total of 77 out of 78 patients in the SST group had Astler Coller B and C disease while the number was 132/138 in the DST group. The mean distance of the tumor from anal verge was 7.6 cm (2.5–15 cm) and 8.0 cm (4–15 cm) in the DST and SST groups, respectively. In the DST group, there were 5 (3.6%) anastomotic failures and 62 (45%) covering stomas compared to 7 (8.9%) anastomotic failures and 51 (65.4%) covering stomas in the SST group. The anastomotic leak rate, though objectively lower in the DST group, did not attain statistical significance (p = 0.12). Covering stoma rates were significantly lower in DST group (p = 0.006). There was 1 death in the DST group due to cardiac causes (unrelated to the anastomosis) and no mortality in the SST group. The LAR and abdominoperineal resection (APR) rates were 40% and 60%, respectively, during 1999–2001. In 2005, these rates were 55% and 45%, respectively.

**Conclusion:**

This study, perhaps the first from India, demonstrates the feasibility of the DST in a country where the incidence of colorectal cancer is increasing. Since the age at presentation is at least a decade younger than the Western world, consideration of sphincter preservation assumes greater significance. The observed improvement of surgical outcomes with DST needs further studies to significantly prove these findings in a population where the tumors at presentation are predominantly Astler Coller Stage B and C.

## Background

Rectal cancer in India is a disease that has been fairly constant in incidence over the last few years. On the other hand, the incidence of colon cancer is increasing [1]. However, the notable trends have been the high incidence of these cancers in the urban population and amongst young Indians. It was estimated that in the year 2001, the incidence of colorectal cancer cases would be 18,427 in males and 13,092 in females [1].

Surgery for low rectal cancer has gradually evolved towards sphincter-preserving operations, egged on by innovations like improved surgical techniques, better suture materials, and most importantly the use of the circular stapler [2-4]. Improved quality of life and comparable recurrence and survival rates have been observed following LAR [5-14]. The introduction of circular staplers has revolutionized gastrointestinal surgery, particularly in colorectal anastomosis to the extent of almost single handedly resulting in the improved number of sphincter-saving operations performed [15]. The technique of SST was fraught with some technical difficulties, most notably, the difficulty of placing a precise purse-string suture in the distal rectum and the problem of the discrepancy in size between the rectum and colon for anastomosis. The introduction of the DST by Knight and Griffin seemed to have resolved these problems and has resulted in a greater number of sphincter-saving operations [16-21]. One concern with the DST was the theoretical risk of increased anastomotic leak at the intersecting staple lines.

While DST is well established for over a decade, data from areas with comparatively lower incidence of colorectal cancers is lacking. In 2003, we implemented DST as a standard procedure for all low anterior resections and prospectively evaluated this technique with regards to the clinical anastomotic leak rates, operative times, blood loss, covering stoma rates, and post operative morbidity. Furthermore, we compared our experience with the outcomes of the previously performed SST (1999 to 2001) in an attempt to make an objective assessment of the perceived and reported advantages of this technique.

## Patients and methods

Between 1999 and 2005, 216 patients with primary adenocarcinoma of the rectum underwent LAR, defined as resection of the rectum with extraperitoneal anastomosis. The data from the year 2002 was excluded owing to administrative changes with consequent lack of protocols and inadequate data maintenance. A comparative analysis was performed between the prospective analysis performed on 138 consecutive LARs performed from January 2003 to December 2005 using the DST and a retrospective analysis performed on 78 patients who underwent LAR from January 1999 to December 2001 using the SST.

Preoperative evaluation included a digital rectal examination, abdominopelvic computed tomography, and colonoscopy to rule out synchronous lesions, to perform a biopsy to confirm diagnosis of rectal cancer, and assess distance of the tumor from the anal verge.

All patients had standard bowel preparation with polyethylene glycol and were given prophylactic antibiotics on induction of anesthesia. As the concept of neoadjuvant treatment was still in the process of being introduced into routine protocols, only 2 patients (in the DST group) received preoperative radiotherapy.

Laparotomy was performed through a low midline incision with the patient in Lloyd-Davies position. The rectum and sigmoid colon was mobilized and inferior mesenteric vessels ligated and divided. Total mesorectal excision was performed. The bowel was divided proximally with a linear cutter. An angled rectal clamp was placed on the rectum distal to the tumor, and the rectal stump was irrigated *per anum *with dilute povidone-iodine. In patients with single-stapled anastomosis, the distal rectum was divided and an over-and-over 1-0 polypropelene (Prolene) purse-string suture was placed. In patients with double-stapled anastomosis, a linear stapler (*Access 55, Ethicon, Johnson & Johnson*) was used to close the distal rectal stump. A purse-string suture was placed in the proximal segment after introducing a well-lubricated anvil, and the anastomosis was completed by passing a circular stapler *per anum (Autosuture/Ethicon, Johnson & Johnson)*. The tissue doughnuts were inspected for completeness before being sent for pathologic examination. The integrity of the anastomosis was verified by air insufflation of the rectum with the pelvis filled with saline.

A single surgical team experienced in rectal cancer surgery performed all operations. A protective covering colostomy was added when tumors were below the peritoneal reflection, in obstructing growths where bowel preparation was considered unsatisfactory, and when completeness of the tissue doughnuts was in doubt.

Anastomotic leaks were defined by the presence of any of the following features: the presence of peritonitis caused by anastomotic dehiscence as noted by lower abdominal tenderness with fever and leucocytosis, the presence of feculent substances and gas from the pelvic drain, the presence of a pelvic abscess with demonstration of anastomotic leak by rectal examination, sigmoidoscopy, or contrast study.

Continuous data are expressed as the mean ± standard deviation. Statistical analysis was performed using the SPSS Version 14.0, Student T test, and Fishers exact test and chi square test for testing statistical significance. A *p *value of < 0.05 was considered as significant.

## Results

The 216 patients undergoing LAR with SST and DST were well matched with regard to age and sex (Table 1). A male predominance was noted in both the groups with the mean age of incidence being as low as 50.3 ± 13.9 years in the DST group. The mean distance of the tumor from the anal verge was 7.6 cm (2.5–15 cm) in the double-stapling group and 8.0 cm (range 4 – 15 cm) in the single-stapling group. The histopathological stage of the two groups was also evenly comparable with the tumors being predominantly modified Dukes C.

**Table 1:** Patient and tumor characteristics

***Characteristic***	***Single staple technique (SST)***	***Double staple technique (DST)***
Patient number	78	138
Age	53.2 ± 13.5 years (21 – 85)	50.3 ± 13.9 years (25 – 75)
Male:Female	56:22	90:48
ASA grade	Data not available	ASA I (94%); ASA II (6%)
Distance of tumor from anal verge	8.0 cm (4 – 15)	7.6 cm (2.5 – 12)
Histopathologic stage of tumors	Dukes A (n = 1) Dukes B1 (n = 10) Dukes B2 (n = 22) Dukes C1 (n = 4) Dukes C2 (n = 41)	Dukes A (n = 5) Dukes B1 (n = 20) Dukes B2 (n = 44) Dukes C1 (n = 8) Dukes C2 (n = 60) Benign (n = 1)

In patients who underwent DST, there were 5 (3.6%) clinical leaks, requiring peritoneal lavage and a colostomy, disconnection with end colostomy, and conservative medical management, respectively. All five patients recovered. There were 62 (45.0%) covering colostomies. There was 1 patient with a (0.7%) tumor positive distal cut margin and 10 had (7.2%) tumor positive circumferential margin. The only mortality seen was due to a cardiac event and unrelated to the disease and the surgical procedure.

In patients who underwent SST, there were 7 (8.9%) clinical leaks and 51 (65.4%) covering colostomies. 4 patients had distal cut margin positive in this group (5.1%). There was no mortality in this group

Blood loss, blood transfusion, and duration of hospital stay have been shown in Tables 2 and 3. The anastomotic dehiscence rates were lower in patients undergoing double-stapling anastomosis than in patients undergoing single-stapling anastomosis but the difference was not significant (p = 0.12). However, the covering colostomy rates were lower in the double stapling group compared to the single stapling group and this difference was statistically significant (p = 0.006) (Table 2).

**Table 2:** Patient outcomes intra operative

***Outcome***	***Single staple technique***	***Double staple technique***
Covering stoma**	51/78 (65.4%)	62/138 (45.0%)
Blood loss	Data not available	300 cc ^# ^(50–1800)
Blood replaced (units/case)	Data not available	0.3 (0–4 units)

*Statistically not significant (p = 0.12)

**Statistically significant difference (p = 0.006)

^# ^Median used due to the large standard deviation

The trend in patients undergoing APR and LAR resection was also evaluated from 1999 to 2005 (Table 4). The percentage of patients with rectal cancer undergoing a sphincter-saving operation has risen since introduction of the double-stapling technique for low anterior resection at our institution in 2003. Patients who underwent LAR using the single-stapling technique from 1999 to 2001 had LAR and APR rates of 40% and 60%, respectively. In 2005, 55% of patients with rectal cancer were treated with LAR and 45% with APR (Table 4).

**Table 3:** Patient outcomes postoperative

***Outcome***	***Single staple technique***	***Double staple technique***
Complications	7 – Anastomotic leaks Data on the other complications not available	16/138 (11.6%) 5 – Anastomotic leaks 5 – wound infections 1 – adhesive intestinal obstruction 5 – sunken stomas
Duration of hospital stay (days)	Data not available	10.7 (4–33)
Distal cut margin positivity	4/78 (5.1%)	1/138 (0.72%)
Circumferential margin status	Data not available	10/138 (7.2%)
Mortality	0	1

**Table 4:** Abdomino-Perineal Resections and Low Anterior Resections for low rectal cancer at the Tata Memorial Hospital: Time trends

***Year***	***Abdominoperineal resection (APR)***	***Low Anterior Resection (LAR)***
1999	60%	40%
2001	60%	40%
2003	50%	50%
2004	46%	54%
2005*	45%	55%

*Statistically not significant compared to figures from 1999 – 2001

## Discussion

The initial apprehension to the sphincter preservation was the ability to obtain sphincter preservation while maintaining oncologic radicality. A recent spate of reports demonstrating equal oncologic outcomes with improved quality of life [2-14] seems to have settled this issue beyond doubt. Numerous advances in colorectal surgery have paved the way for the increase in sphincter-saving operations. Firstly, the importance of the mesorectum in local recurrence was recognized [22-24]. Recurrence rates of < 5% have been reported with a distal margin of < 2 cm, provided the mesorectum could be completely excised [25]. Second, the introduction of circular stapling devices has facilitated colorectal anastomoses close to the dentate line. Third, the development of the DST further increased the performance of sphincter-saving operations for low rectal cancer [16]. These innovations have resulted in reported rates of APR to the tune of 23% and 27.8% for tumors located < 5 cm and 6 cm, respectively, from the anal verge [2,24]. A distal margin of < 1 cm has been shown to be acceptable in distal rectal cancers [26,27], and APR is now reserved only for patients with very low tumors where an adequate margin cannot be obtained or the anastomosis is not feasible [3,24], or in patients with a poor sphincter tone – inherent, or due to destruction by the malignancy.

In the present study, we observed that the DST for LAR is associated with fewer complications, a lower anastomotic leak rate, and significantly fewer covering stomas than the SST.

Anastomotic dehiscence is a major concern following LAR for rectal cancer owing to the high morbidity and mortality [28]. Previous studies on LAR using the DST have reported acceptable anastomotic leak rates [21,29-32]. Table 5 shows some of the larger reported series of DST with their leak rates [21,29-32]. The clinical leak rate of 3.6% using the DST in the present study confirms these results. Few studies have compared clinical leak rates in patients undergoing LAR using SST and DST. Moritz *et al. *showed a trend towards a lower clinical leak rate in patients undergoing DST but this finding was not statistically significant [33].

**Table 5:** Comparison of anastomotic leak rates

***Study***	***Year***	***Number of patients***	***Leak rates***
Baran et al. [21]	1992	104	2.7%
Moran et al. [29]	1992	55	9.0%
Redmond et al. [30]	1993	111	2.8%
Laxamana et al. [31]	1995	189	7.3%
Kanellos et al. [32]	2004	93	9.7%
TMH study	2003–2005	138	3.6%

Concerns about anastomotic failure at intersecting staple lines appear to be unwarranted based on our findings. Several explanations might account for our improved outcomes using the DST. Probably, the most important factor that accounts for a better outcome after the use of DST is the ease of performance of the anastomosis. Since this procedure is being performed for low rectal lesions, the most daunting task is to be able to divide the bowel close to the levator ani muscles and perform a deep anastomosis within the confines of the pelvic cavity. The available area is further reduced in a male pelvis (our data records a higher number of treated male patients) and in obese patients. Moreover, bulky lesions, as seen in most Asian countries (perhaps due to lack of awareness and subsequent delayed presentation) render division of the bowel distal to the tumour very difficult. Furthermore, this technique eliminates the technical difficulty of placement of a distal purse-string suture. Other advantages of double-stapled anastomosis are minimization of fecal contamination and spillage of tumor cells from the rectum that may occur during single-stapling anastomosis [17,29]. Another possible advantage might be that this technique virtually eliminates inter-surgeon variability because of the mechanized nature of the linear stapler. Using this technique, it is plausible that the distal doughnut will be more precise independent of the surgeon performing the anastomosis. Use of a linear stapler to approximate the distal rectum may therefore provide more consistent results. Further, no studies have demonstrated an increased recurrence at the staple line following DST [29,31]. Lastly, increasing familiarity with the same team performing the procedure over time probably does have a role to play in improved results. As regards this reason in the present study, it is worthwhile noting that the SST was also a technique practiced routinely by the same team prior to 2001–2002.

Previous reports have shown decreased covering stoma rates in patients undergoing LAR with SST when compared with hand-sutured anastomosis [34]. We now report that fewer covering stomas are required following LAR using the DST compared to the SST. While a reduction in covering stoma rates can be due to a number of unrelated factors, the operating surgeon's subjective sense of comfort remains a crucial factor. It appears that a heightened sense of security regarding the integrity of the anastomosis, despite a new technique being attempted when one would expect a tendency to err on the side of safety, was at least in part responsible for reduced covering stoma rates following the DST compared to the SST. Consequently, stoma-related complications are also minimized [35]. In a country where stoma care facilities are still in the developmental stage and patients can yet face problems of social acceptance, our experience assumes significance. Also, the absence of covering protective stomas reduces the cost of its maintenance and future closure by an additional surgical procedure.

Conventional DST is mainly performed for tumors > 6 cm from the anal verge [31,36,37]. We have observed a rise in the percentage of patients undergoing sphincter-saving operations since the introduction of the DST. While increasing experience can certainly account for increasing number of sphincter preserving procedures, we believe that DST is also responsible for the improved deeper access and division of the distal rectum using the linear stapler, particularly when division occurs below the peritoneal reflection. However, our present results from the non-randomized nature of this study cannot fully substantiate this belief.

In India, with approximately half the incidence of rectal cancer compared to Western data [1], there is a lack of data on stapling techniques following resection for low rectal cancer. Rectal cancer, in India, is more common than colon cancer and trends show a high incidence among young Indians [1], a finding that can neither be explained by heredity nor traditional diet. The high incidence in younger patients makes sphincter preservation a more desired option in this subgroup.

## Conclusion

This study represents the first large Indian experience comparing stapling techniques for LAR of rectal cancer. Our findings have opened the avenue for a more serious consideration of the use of DST in a population where cost effectiveness and bulky tumors at presentation [38] pose a major challenge to surgeons causing them to have apprehensions to the use of this technique, its feasibility and safety. However, larger numbers of patients are required to obtain statistically significant results and establish this technique as standard of care.

## Competing interests

The author(s) declare that there are no competing interestes

## Authors' contributions

**SVS **– Conceptualization of design, data research, data analysis and preparation of the manuscript

**RS **– Conceptualization of design, Data collection

**GB **– Data research and preparation of the manuscript

**AK **– Statistical analysis and preparation of manuscript

**SW **– Data Research and preparation of manuscript

**FG **– Data collection and preparation of manuscript

**SA **– Data collection and preparation of manuscript

PJS – Data research, data analysis and preparation of the manuscript

## References

[B1] Mohandas KM, Desai DC (1999). Epidemiology of digestive tract cancers in India. V. Large and small bowel. Indian J Gastroenterol.

[B2] Heald RJ, Smedh RK, Kald A, Sexton R, Moran BJ (1997). Abdominoperineal excision of the rectum – an endangered operation. Norman Nigro Lectureship. Dis Colon Rectum.

[B3] Ota DM, Jacobs L, Kuvshinoff B (2002). Rectal cancer: the sphincter-sparing approach. Surg Clin N Am.

[B4] Di Betta E, D'Hoore A, Filez L, Penninckx F (2003). Sphincter saving rectum resection is the standard procedure for low rectal cancer. Int J Colorectal Dis.

[B5] Amato A, Pescatori M, Butti A (1991). Local recurrence following abdominoperineal excision and anterior resection for rectal cancer. Dis Colon Rectum.

[B6] Dixon AR, Maxwell WA, Thornton, Holmes J (1991). Carcinoma of the rectum: a 10-year experience. Br J Surg.

[B7] Fegiz G, Indinnimeo M, Gozzo P, Del Grande E, Cataldi S, Brozzetti S (1994). Low rectal cancer--What is the choice?. Dis Colon Rectum.

[B8] Bokey EL, Chapuis PH, Fung C, Hughes WJ, Koorey SG, Brewer D, Newland RC (1995). Postoperative morbidity and mortality following resection of the colon and rectum for cancer. Dis Colon Rectum.

[B9] Lavery IC, Lopez-Kostner F, Fazio VW, Fernandez-Martin M, Milsom JW, Church JM (1997). Chances of cure are not compromised with sphincter-saving procedures for cancer of the lower third of the rectum. Surgery.

[B10] Rullier E, Laurent C, Carles J, Saric J, Michel P, Parneix M (1997). Local recurrence of low rectal cancer after abdominoperineal and anterior resection. Br J Surg.

[B11] Topal B, Penninckx F, Kaufman L, Filez L, Aerts R, Ectors N, Kerremans R (1998). Outcome after 'curative' surgery for carcinoma of the lower one-third of the rectum. Br J Surg.

[B12] Jatzko GR, Jagoditsch M, Lisborg PH, Denk H, Klimpfinger M, Stettner HM (1999). Long-term resutls of radical surgery for rectal cancer: multivariate analysis of prognostic factors including survival and local recurrence. Eur J Surg Oncol.

[B13] Secco GB, Fardelli R, Campora E, Baldi E, Bonfante P, Ferraris R (1999). Local control after curative surgery for cancer of the extraperitoneal rectum. Oncology.

[B14] Nakagoe T, Ishikawa H, Sawai T, Tsuji T, Tanaka K, Hidaka S, Nanashima A, Yamaguchi H, Yasutake T (2004). Survival and recurrence after a sphincter-saving resection and abdominoperineal resection for adenocarcinoma of the rectum at or below the peritoneal reflection: a multivariate analysis. Surg Today.

[B15] McGinn FP, Gartell PC, Clifford PC, Brunton FJ (1985). Staples or sutures for low colorectal anastomoses: a prospective randomized trial. Br J Surg.

[B16] Knight CD, Griffin FD (1980). An improved technique for low anterior resection using the EEA stapler. Surgery.

[B17] Cohen Z, Myers E, Langer B, Taylor B, Railton RH, Jamieson C (1983). Double stapling technique for low anterior resection. Dis Colon Rectum.

[B18] Griffen FD, Knight CD Low rectal anastomosis with the EEA stapler. Surgical Rounds.

[B19] Feinberg SM, Parker F, Cohen Z (1986). The double stapling technique for low anterior resections of rectal carcinoma. Dis Colon Rectum.

[B20] Picciocchi A, D'Ugo DM, Durastante V, Cardillo G (1988). Double stapling technique for low colorectal anastomosis after anterior resection for rectal cancer. Int Surg.

[B21] Baran JJ, Goldstein SD, Resnik AM (1992). The double-stapling technique in colorectal anastomosis. Am Surg.

[B22] Heald RJ, Ryall RD (1986). Recurrence and survival after total mesorectal excision for rectal cancer. Lancet.

[B23] McCall JL (1997). Total mesorectal excision: evaluating the evidence. Aust N Z J Surg.

[B24] Law WL, Chu KW (2001). Impact of total mesorectal excision on the results of surgery of distal rectal cancer. Br J Surg.

[B25] Karanjia ND, Schache DJ, North WR, Heald RJ (1990). 'Close shave' in anterior resection. Br J Surg.

[B26] Andreola S, Leo E, Belli F, Lavarino C, Buafalino R, Tomasic G, Baldini MT, Valvo F, Navarria P, Lombardi F (1997). Distal intramural spread in adenocarcinoma of the lower third of the rectum treated with total rectal resection and coloanal anastomosis. Dis Colon Rectum.

[B27] Vernava AM, Moran M, Rothenberger DA, Wong WD (1992). A prospective evaluation of distal margins in carcinoma of the rectum. Surg Gynecol Obstet.

[B28] Fielding LP, Stewart-Brown S, Hittinger R, Blesovsky L (1984). Covering stoma for elective anterior resection of the rectum: an outmoded operation?. Am J Surg.

[B29] Moran BJ, Blenkinsop J, Finnis D (1992). Local recurrence after anterior resection for rectal cancer using a double stapling technique. Br J Surg.

[B30] Redmond HP, Austin OMB, Clery AP, Deasy JM (1993). Safety of double-stapled anastomosis in low anterior resection. Br J Surg.

[B31] Laxamana A, Solomon MJ, Cohen Z, Feinberg SM, Stern HS, McLeod RS (1995). Long-term results of anterior resection using the double-stapling technique. Dis Colon Rectum.

[B32] Kanellos I, Vasiliadis K, Angelopoulos S, Tsachalis T, Pramateftakis MG, Mantzoros I, Betsis D (2004). Anastomotic leakage following anterior resection for rectal cancer. Tech Coloproctol.

[B33] Moritz E, Achleitner D, Holbling N, Miller K, Speil T, Weber F (1991). Single vs double stapling technique in colorectal surgery. Dis Colon Rectum.

[B34] Heald RJ (1980). Towards fewer colostomies--the impact of circular stapling devices on the surgery of rectal cancer in a district hospital. Br J Surg.

[B35] Graffner H, Fredlund P, Olson S, Oscarson J, Petersson BG (1983). Protective colostomy in low anterior resection of the rectum using the EEA stapling instrument. A randomized study. Dis Colon Rectum.

[B36] Fu CG, Muto T, Masaki T (1997). Results of the double stapling procedure in colorectal surgery. Surg Today.

[B37] Griffen FD, Knight CD, Knight CD (1992). Results of the double stapling procedure in pelvic surgery. World J Surg.

[B38] Deo SV, Shukla NK, Srinivas G, Mohanti BK, Raina V, Sharma A, Rath GK (2001). Colorectal cancers – experience at a regional cancer center in India. Trop Gastroenterol.

